# Is There an Association between Breastfeeding and Dental Caries among Three-Year-Old Australian Aboriginal Children?

**DOI:** 10.3390/nu11112811

**Published:** 2019-11-18

**Authors:** Dandara G. Haag, Lisa M. Jamieson, Joanne Hedges, Lisa G. Smithers

**Affiliations:** 1 Indigenous Oral Health Unit, Australian Research Centre for Population Oral Health, The University of Adelaide, Adelaide, SA 5005, Australia; dandara.haag@adelaide.edu.au (D.G.H.); lisa.jamieson@adelaide.edu.au (L.M.J.); joanne.hedges@adelaide.edu.au (J.H.); 2 School of Public Health, The University of Adelaide, Adelaide, SA 5005, Australia

**Keywords:** breastfeeding, caries, childhood, Aboriginal

## Abstract

An unresolved question about breastfeeding is its effect on caries, in particular, early childhood caries (ECC). In secondary analyses of data from an ECC intervention, we describe breastfeeding among Aboriginal children and associations between breastfeeding and ECC. Breastfeeding (duration and exclusivity to six months) was grouped into mutually exclusive categories. ECC was observed by a calibrated dental professional. Outcomes were prevalence of ECC (% decayed, missing, and filled teeth in the primary dentition (% dmft>0)) and caries severity (mean number of decayed, missing, and filled surfaces (mean dmfs)) in children aged three years. Analyses were adjusted for confounding. Multiple imputation was undertaken for missing information. Of 307 participants, 29.3% were never breastfed, 17.9% exclusively breastfed to six months, and 9.3% breastfed >24 months. Breastfeeding >24 months was associated with higher caries prevalence (adjusted prevalence ratio (PR_a_) 2.06 (95%CI 1.35, 3.13, *p*-value = 0.001) and mean dmfs (5.22 (95% CI 2.06, 8.38, *p*-value = 0.001), compared with children never breastfed. Exclusive breastfeeding to six months with breastfeeding <24 months was associated with 1.45 higher caries prevalence (95% CI –0.92, 2.30, *p*-value = 0.114) and mean dmfs 2.04 (−0.62, 4.71, *p*-value = 0.132), compared with never breastfeeding. The findings are similar to observational studies on breastfeeding and caries but not with randomized controlled trials of breastfeeding interventions. Despite attending to potential biases, inconsistencies with trial evidence raises concerns about the ability to identify causal effects of breastfeeding in observational research.

## 1. Introduction

Breastfeeding has many well-established benefits for both mother and baby, and is recommended by governments, scientific, and health institutions all over the world. The World Health Organization (WHO) recommends that infants are exclusively breastfed to age six months, and that breastfeeding continues to two years of age [1]. Individual countries may vary slightly in their recommendations, for example, the Australian government recommends exclusive breastfeeding to six months and continued breastfeeding to up to and beyond one year [2]. Despite these recommendations, there is little recent evidence on adherence to these guidelines by marginalized communities, such as Aboriginal and Torres Strait Islander peoples. Breastfeeding is culturally supported by Aboriginal and Torres Strait Islanders and is encouraged by local Aboriginal Community Controlled Health Service workers and midwifes.

An unresolved question about breastfeeding is its effect on caries, in particular, early childhood caries (ECC). ECC is defined as the presence of one or more decayed (cavitated or not), missing (due to dental caries), or filled tooth surfaces in any primary tooth among children aged <6 years old [3]. In recent years, there have been a number of systematic reviews reporting the effect of breastfeeding on ECC that have arrived at slightly differing conclusions. One of the first systematic reviews published by Tham et al. [4] included 63 papers published up until 2014. Tham et al. concluded that breastfeeding protected against ECC but breastfeeding beyond 12 months of age increased the risk of ECC. Cui et al. suggested similar findings from a search up to 2015 that included 35 studies but called for more studies to strengthen the evidence [5]. While it seems that breastfeeding in the early postnatal period is protective, the time at which the association reverses and breastfeeding becomes a risk factor for ECC is unclear. Earlier this year, Moynihan et al. published a systematic review that investigated this further [6], where they examined studies that compared the effects of breastfeeding ≥12 months compared to <12 months on ECC, and studies of breastfeeding ≥24 months compared to <24 months. For the 21 studies comparing breastfeeding <12 vs. ≥12 months, a null association was observed. The evidence from the eight studies comparing breastfeeding ≥24 months compared to <24 months varied widely, with some studies reporting strong positive associations with ECC, but studies were too small to generate a precise effect estimate and a null association was inferred.

We previously collected breastfeeding and ECC information from Australian Aboriginal children and their families who participated in a randomized controlled trial of an early childhood caries intervention. In Australia, oral health inequalities between Aboriginal and non-Aboriginal people start to emerge in childhood. According to the Australian National Child Oral Health Study (NCOHS) conducted between 2012 and 2014, the prevalence of untreated dental caries in the primary dentition among Indigenous children aged 5–10 years old was 44% compared to 26% among their non-Indigenous counterparts [7]. This difference represents a 70% increase in the prevalence of untreated dental caries, and it was larger than the inequalities observed according to other important sociodemographic factors, such as household income, parental education, and residential location. Furthermore, not only the prevalence of untreated dental caries is higher among Indigenous children, but they also have more severe disease. Indigenous Australian children aged 5 to 10 years old have, on average, almost three times the mean number of decayed tooth surfaces observed among their non-Indigenous counterparts (3.4 vs. 1.2). This means that Indigenous children are more likely to be exposed to the consequences of dental caries, such as dental pain, treatment under general anesthesia, poorer concentration in a classroom setting, and higher school absenteeism. Therefore, understanding potential drivers of dental caries at an early age in this population is the first step to developing culturally appropriate interventions to manage dental disease in its early stages, and to instituting effective preventive measures to improve oral health in adulthood. Here, we conduct a secondary (observational) analyses of these data with two aims: (1) To describe patterns of breastfeeding among Australian Aboriginal children, and (2) to examine the association between different categories of breastfeeding on three-year-old ECC outcomes.

## 2. Materials and Methods 

### 2.1. Participants and Study Design

The current study comprises a secondary analysis using data from an early childhood caries intervention that involved 448 women who identified as being pregnant with an Aboriginal and/or Torres Strait Islander child between February 2010 and May 2011 [8]. 

Recruitment was mainly through the antenatal clinics at South Australian hospitals and through Aboriginal Community Controlled Health Organizations. The study was a 2-arm parallel, outcome assessor-blinded, randomized controlled trial that aimed to assess if an intervention involving dental care to mothers during pregnancy, application of fluoride varnish to the teeth of children, anticipatory guidance, and motivational interviewing reduced prevalence of dental disease among Aboriginal children in South Australia at follow-up ages of 24 and 36 months [9,10]. The intervention took place during pregnancy and when children were aged 6, 12, and 18 months for the early intervention group, and when children were aged 24, 30, and 36 months for the delayed intervention group. Three-year follow-up data were collected from November 2014 to February 2016. All information was collected at participants’ households or at their ACCHO. Children with any information collected at baseline, 2,- and 3-year-old follow-up were included in the study (*n* = 307). 

All participants provided written informed consent. Both the original trial and the follow-up studies were conducted in accordance with the Declaration of Helsinki, and the protocol was approved by the Ethics Committee of The University of Adelaide Human Research Ethics Committee (Project code: H-057-2010), the Aboriginal Health Council of South Australia (Project code: 04-09-362), the South Australian Department for Health, including the human research ethics committees of participating South Australian hospitals (Flinders Medical Centre (Project code: 435-10), Lyell McEwin Hospital (Project code: 2010-160), and Women’s and Children’s Hospital (Project code: REC2322/11/13)).

### 2.2. Breastfeeding (Exposure)

Breastfeeding information was collected when children were aged 24 months by asking mothers the duration of breastfeeding and the period of exclusive breastfeeding. Patterns of breastfeeding were categorized according to two definitions based on previous studies examining the effect of breastfeeding on dental caries (definition A) [4,6] and following the WHO recommendations (definition B) [1]. Definition A allows us to compare the risks of ECC for breastfeeding over 12 months with other durations of breastfeeding, whereas definition B aligns with the WHO’s international breastfeeding recommendations. Definition A consisted of four categories including children who were never breastfed, breastfed for <12 months, breastfed for 12–23 months, and breastfed for 24 months or longer. Definition B consisted of the following four categories of breastfeeding; never breastfed, breastfed for <24 months with exclusive breastfeeding until 6 months, breastfed for <24 months with no exclusive breastfeeding until 6 months, and breastfed for 24 months or longer.

### 2.3. Oral Health (Outcomes)

The primary outcome was the prevalence of ECC, which is the proportion of children with ≥1 decayed teeth (with or without a break in the tooth surface), ≥1 missing teeth due to dental caries, and ≥1 filled teeth) at 3 years of age (% dmft>0). The primary dentition comprises 20 teeth, which normally erupt in the oral cavity between 6 and 24 months of age. Dental decay was considered since its initial stages, which consists of white areas on the surface of the tooth. We also examined the severity of ECC by assessing the mean number of tooth surfaces with decayed, missing, and filled surfaces (mean dmfs). Oral examinations were conducted by calibrated and masked examiners following a standardized protocol to record dental disease experience. Children were examined in the knee-to-knee position on their mother’s lap, as is considered appropriate for this age group. Teeth were dried with cotton pads before examination. The light source was a fiber-optic light and standard infection control procedures were followed. Only visual criteria were used to assess diagnoses. Any children identified as having untreated dental decay were referred to the South Australian Dental Service (SADS).

### 2.4. Confounding

Confounding factors included a range of variables that share a causal association with both exposures and outcomes and were assessed at baseline. These included smoking and alcohol consumption in pregnancy (yes/no), child sex, main source of household income (job vs. government social welfare support), maternal education (secondary school or less vs. trade/technical and university), parity (first time mother vs. others), maternal age (continuous), maternal ethnicity (Aboriginal vs. non-Aboriginal), and a national indicator of socioeconomic position calculated from residential postcode (the index of relative socioeconomic advantage and disadvantage (IRSAD) [11]. Additionally, we adjusted for randomization group because breastfeeding occurred during the intervention, even though there were no differences in breastfeeding duration between the treatment groups.

### 2.5. Statistical Analysis

In order to address potential bias due to missing data, we undertook multiple imputation by chained equations under the assumption that data were missing at random conditional on the observed data [12]. Twenty imputed datasets were generated from imputation models containing all the variables included in the final regression analyses. Imputed values were generated for smoking in pregnancy (missing *n* = 1), alcohol consumption in pregnancy (missing *n* = 1), main source of household income at baseline (missing *n* = 1), IRSAD (missing *n* = 3), breastfeeding duration (missing *n* = 3), exclusive breastfeeding duration (missing *n* = 9), and mean number of tooth surfaces with dental caries (missing *n* = 1). All other variables were fully observed. Analyses were conducted by combining all imputed datasets following Robin’s rules on a final sample of *n* = 307 children who had some information collected at baseline, 2-, and 3-year-old follow-up.

Descriptive statistics were computed for the response sample, complete cases, and imputed sample, and included the distribution of participants according to exposures (breastfeeding), confounding factors (listed above), and outcomes (prevalence of ECC and mean dmfs). Generalized linear models with a log-Poisson link function and robust standard errors were used to estimate the prevalence ratios [13] and their 95% confidence intervals (CI) of dental caries according to breastfeeding patterns (definitions A and B). Next, adjusted PRs and their respective 95% CIs were assessed after the inclusion of the abovementioned confounding variables in the models. Unadjusted and adjusted and linear regressions were used to estimate the association between breastfeeding patterns and mean dmfs. 

Analyses were carried out using STATA 15.0.

## 3. Results

The sample consisted of 307 children who had some information collected at baseline, 2-, and 3-year-old follow-up. Sample characteristics are shown in Table 1. Most children were born to mothers who identified as Aboriginal (82.7%). On average, mothers were 24.7 years old at baseline. Just over half of the children (52.1%) were boys and 39.4% were the first child. Four in five children (82.4%) lived in households where government support was the main source of income and over two-thirds of mothers (71.7%) had high school or less educational attainment. Over half of the children (55.3%) lived in the most disadvantaged IRSAD quintile. Smoking in pregnancy was observed among half of the sample, whereas 10.8% of mothers reported alcohol consumption during pregnancy. One in three children were never breastfed (29.2%), whereas half of the sample (49.9%) were breastfed for less than 12 months. The proportion of children breastfed for 12–23 months and 24 or more months was 11.5% and 9.3%, respectively. One in four children were exclusively breastfed until six months (23.8%) (data not shown). At three years of age, one-third had experienced dental caries, and the mean number of decayed, missed, or filled tooth surfaces was 3.2 (Table 1).

**Figure 1:**
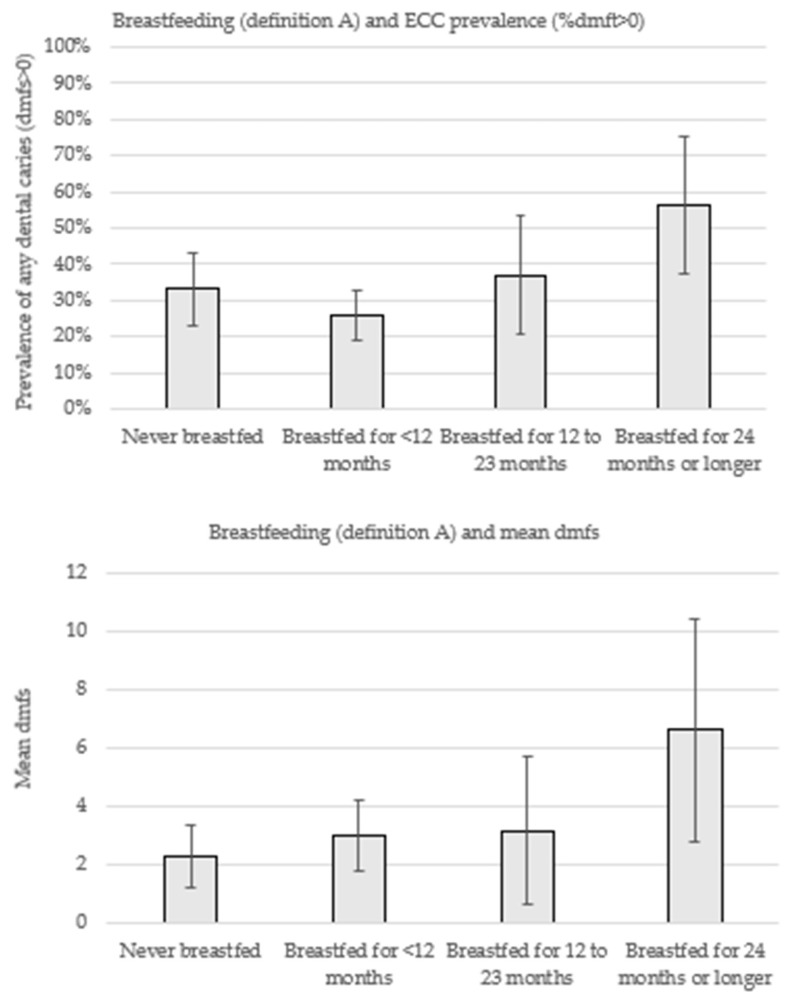
Prevalence of early childhood caries (ECC) and mean number of decayed, missing, and filled teeth (mean dmfs) according to breastfeeding patterns (definition A).

Figure 1 displays the prevalence of dental caries experience (% dmft>0) and mean dmfs according to breastfeeding patterns (definition A). In comparison with children who were never breastfed, the prevalence of ECC was lower among those who were breastfed <12 months (33.1% vs. 25.7%), although both groups exhibited a similar mean dmfs (2.3 vs. 3.0). Over two-thirds of the children breastfed for 12 to 23 months had dental caries (37.0%). The highest prevalence (56.4%) and severity (6.6 mean dmfs) of disease was observed among children breastfed for 24 months.

**Figure 2:**
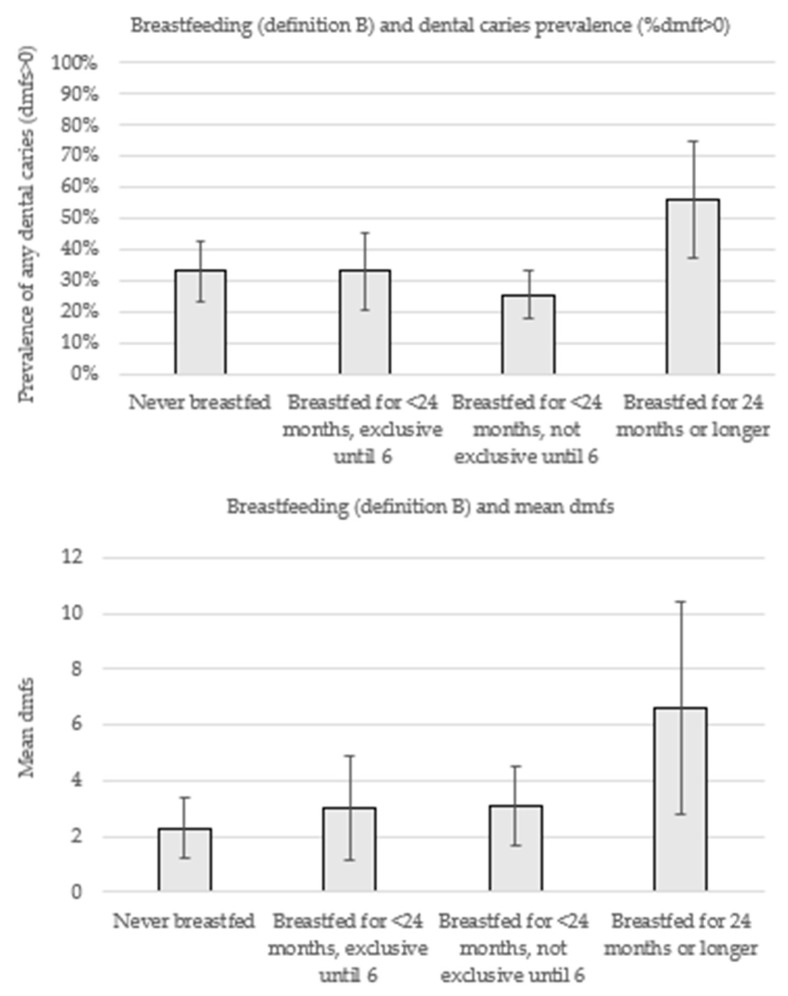
Prevalence of early childhood caries (ECC) and mean number of decayed, missing, and filled teeth (mean dmfs) according to breastfeeding patterns (definition B).

Figure 2 displays the prevalence of dental caries experience (% dmft>0) and mean dmfs according to breastfeeding patterns (definition B). While a higher prevalence of dental caries was observed among children breastfed for less than 24 months with exclusive breastfeeding until six months (33.1%) in comparison with those breastfed for the same duration but without exclusive breastfeeding (25.7%), both groups exhibited a similar mean dmfs (3.0 vs. 3.1).

Table 2 displays the unadjusted and adjusted associations between breastfeeding patterns (definitions A and B) with % dmft>0 and mean dmfs. For definition A, the point estimate for prevalence of % dmft>0 was 6% lower among children who were breastfeeding <12 months compared with no breastfeeding (PR = 0.94 (95%CI 0.63; 1.41)). Breastfeeding for 12 to 23 months and breastfeeding for two years or more were associated with a 47% (PR = 1.47 (95%CI 0.85; 2.54)) and two-fold higher ECC prevalence (PR = 2.06 (95%CI 1.35; 3.13)), respectively. In comparison with children who were never breastfed, adjusted models for the mean dmfs showed higher dmfs among children breastfed for less than 12 months (β = 1.31 (95%CI-0.66; 3.30)), those breastfed for 12 to 13 months (β = 2.24 (95%CI –0.74; 5.24)), and among those breastfed for 24 months or longer (β = 5.22 (95%CI 2.06; 8.39). For definition B, breastfeeding up to 23 months and exclusively until six months was associated with a 45% (PR = 1.45 (95%CI 0.92; 2.30)) higher ECC prevalence and a higher dmfs (β = 2.04 (95%CI –0.62; 4.71)).

## 4. Discussion

The results presented here are secondary, observational analyses from a sample of Aboriginal families in South Australia who were enrolled in an early childhood caries intervention. They show that that around one-third of children are never breastfed, half are breastfed for less than 12 months, and one-fifth are breastfed for longer than 12 months (breastfeeding definition A). Applying the WHO breastfeeding recommendations in breastfeeding definition B shows that almost one-fifth of children are breastfed exclusively for six months. For our aim of investigating observational associations between breastfeeding and oral health, these results suggest that breastfeeding for 24 months or longer is associated with twice the prevalence of ECC and four-fold higher severity of caries (indicated by mean dmfs). Both duration and exclusivity of breastfeeding may be important, but evidence from the current study is stronger for the duration of breastfeeding. Evidence for duration comes from the breastfeeding pattern A results, where the adjusted point estimates suggest monotonically increasing risks of both % dmft>0 and dmfs from 12–23 and then ≥24 months of breastfeeding. Breastfeeding pattern B provides some suggestive observational evidence of an increased risk of caries with exclusive breastfeeding to six months, although there are major challenges with disentangling duration and exclusivity (discussed further below). Additionally, the modest sample size has likely contributed to imprecision in estimating these effects. Notably, this sample has poor oral health with 25% prevalence of ECC even though children were only three years old, which has contributed to why we have been able to detect these associations at such a young age.

It is important to emphasize that any potential effects of breastfeeding on caries can be mitigated by better oral hygiene and access to oral healthcare, and these should be the targets of public policy, particularly since oral health is a major cause of problems (such as hospitalizations, extractions) in children. Maternal-child health nurses encourage breastfeeding and with appropriate training they might present an opportunity for teaching caregivers to begin clean deciduous teeth as soon as they erupt. The promotion of wide-spread water fluoridation is also important in reducing oral health inequalities, particularly between Indigenous and non-Indigenous Australian children [7].

Compared with Australian national statistics on breastfeeding the prevalence, data reported here appear lower. For example, national data indicate that around 80% of Aboriginal children were ever breastfed [14], whereas 70% of children in the current study were reportedly ever breastfed. The national data were collected in 2014–2015, at a similar time to our study, so temporal shifts in breastfeeding cannot explain this discrepancy. This is probably not due to selection bias as, compared with birth registry data, we enrolled around half of all eligible Aboriginal children born in the state over the recruitment period, and families who were not enrolled or dropped out are likely to be more marginalized. While it is difficult to pinpoint exactly why we found lower breastfeeding outcomes compared with national averages, we speculate that this may be because our participants are more disadvantaged than families included in the national data. The high level of disadvantage is indicated in Table 1, where half of the participants in our study are in the most disadvantaged quintile for socioeconomic position (on a national scale), half smoked during pregnancy, and about 80% received government income support. The state of South Australia has a more disadvantaged socioeconomic profile than other mainland Australian states. However, many other (non-socioeconomic) issues around breastfeeding should also be considered. Such factors might include mothers being very time-poor, impediments to breastfeeding due to mental health problems (postnatal depression, anxiety), low self-confidence in their ability to breastfeed, and lack of partner support. Taking a strengths-based view, since only one-third of women did not breastfeed, this indicates this is an area of cultural strength. Contributors to breastfeeding success are other key women involved in perinatal care and social networks of new mothers’, which may improve self-confidence in their ability to successfully initiate and maintain breastfeeding for longer. Social context and culturally safe birthing programs are important influences on breastfeeding among Aboriginal women [15,16].

The debate about whether breastfeeding has a causal role in ECC remains, with discussions on the evidence from the three systematic reviews on this topic vary. Our results appear to confirm concepts raised in previous systematic reviews that the risk of ECC increases with breastfeeding beyond 12 months and possibly with exclusive breastfeeding [4,6]. We were surprised to find suggestive evidence that exclusive breastfeeding to six months (among children breastfed for less than 24 months) was linked to higher caries, as first teeth only erupt around six months. It is possible that the exclusive breastfeeding to six months is an indicator of other related factors, such as mothers being more committed to breastfeeding and more likely to breastfeed for longer. Support for this idea comes from the fact that the duration of breastfeeding among children exclusively breastfed to six months was 2.5 times longer than the duration observed among children who were not exclusively breastfed to six months (10 vs. 4 months). It is clearly difficult to separate out effects of exclusive breastfeeding from the effect of duration of breastfeeding in observational research, and for this reason we look to the literature for evidence from specific breastfeeding randomized controlled trials (RCTs). The standout in this field is the large Promotion of Breastfeeding Intervention Trial (PROBIT), trial which is a cluster-RCT where intervention hospitals implemented the baby-friendly hospital initiative, and this resulted in increased breastfeeding duration and exclusivity [13]. Follow up of caries data in the PROBIT participants indicated no differences in caries outcomes between the groups, despite having more women in the intervention group breastfeeding for longer durations and higher proportions of women exclusively breastfeeding to six months of age [17]. Another cluster RCT of an intervention that increased exclusive breastfeeding to six months conducted in Uganda also indicated no effect of caries outcomes at five years of age [18]. Observational research on breastfeeding is notoriously confounded and one way forward might be to apply different study designs that can help overcome such problems, as has been done for the effects of breastfeeding on intelligence quotient and obesity [19].

We approached this study with causal inference in mind. With respect to study validity, we tried to minimize confounding by adjusting for multiple variables that were identified through content knowledge and literature. One issue relevant to this field is whether to adjust for confounding by other aspects of the child’s diet such as sugary foods and beverages. In causal inference theory, confounding is considered to be a common cause of the exposure and outcome [20]. While there is no doubt that the sugar content of the diet has a direct effect on caries, it is not a direct cause of initiating breastfeeding. Any reduction in breastfeeding will lead to consumption of other foods and therefore sugary foods may be a mediator on the breastfeeding → diet → caries pathway. Factors further back in the causal chain, such as parental education, influence breastfeeding and diet, a phenomenon occasionally referred to as M-bias in the epidemiological literature. Our approach was to adjust for many of these more distal factors to block the influence of these cofounders. It is also worth noting that the participants were involved in a health promotion trial and therefore their breastfeeding behaviors may have been different had they not been involved in the trial. We used multiple imputation to address bias due to missing information, which is uncommon in this field [4,6]. Some effect estimates have wide confidence intervals which is likely due to the sample size, which was limited to the original trial. Increasing the sample size was not possible because the study is estimated to have enrolled around half of the local Aboriginal and Torres Strait Islander children in the state of South Australia at the time of recruitment.

## 5. Conclusions

There is room to improve breastfeeding prevalence, duration, and exclusivity among mothers of Aboriginal children in South Australia, and this could have flow on effects of improving other health outcomes. Regarding ECC, the observational analyses presented here suggest that breastfeeding for longer and more exclusively may be linked to poorer outcomes. Despite attempting to address sources of bias, doubt is raised about interpreting this observational research as causal. 

## Figures and Tables

**Table 1:** Characteristics of the study sample.

	Response Sample %	Complete Cases % (*n* = 292)	Imputed Sample % (*n* = 307)
Exposure: Breastfeeding patterns			
Breastfeeding pattern A			
Never breastfed	29.3	29.8	29.2
Breastfed for <12 months	49.8	51.7	49.9
Breastfed for 12 to 23 months	11.4	11.3	11.5
Breastfed for 24 months or more	9.5	7.2	9.3
Breastfeeding pattern B (WHO recommendations)			
Never breastfed	29.3	29.8	29.2
Breastfed for <24 months, exclusively until 6	17.9	18.5	18.2
Breastfed for <24 months, not exclusively until 6	43.3	44.5	43.2
Breastfed for 24 months or more	9.5	7.2	9.3
Outcomes at child age 3 years			
Any decayed, missed or filled tooth (% dmft>0)	32.0	31.9	32.1
Mean number of decayed, missed or filled tooth surfaces (mean dmfs)	3.1	3.2	3.2
Potential confounding factors			
Child sex (male)	52.1	52.1	52.1
Mean maternal age at childbirth	24.7	24.6	24.7
Main household income source			
Job	17.6	18.2	17.6
Government support	82.4	81.9	82.4
Maternal education at birth			
High school or less	71.7	71.2	71.7
TAFE or university	28.3	28.8	28.3
Index of Relative Socioeconomic Advantage and Disadvantage			
1^st^ quintile (most disadvantaged)	55.4	55.1	55.3
2^nd^ quintile	17.3	16.8	17.4
3^rd^ quintile	21.1	23.6	21.0
4^th^ quintile	2.6	2.7	2.6
5^th^ quintile (most advantaged)	1.6	1.7	1.6
Alcohol consumption in pregnancy (yes)	10.8	10.6	10.8
Smoking in pregnancy (yes)	50.1	50.7	50.0
Parity (first child)	39.4	40.1	39.4
Maternal Ethnicity (Aboriginal and/or Torres Strait Islander)	82.7	82.5	82.7
Child received the intervention (early)	49.5	49.0	49.5

WHO: World Health Organization.

**Table 2:** Associations between breastfeeding patterns with dental caries prevalence and severity.

	Any Decayed, Missed or Filled Tooth (% dmft>0)		Number of Decayed, Missed or Filled Tooth Surfaces (Mean dmfs)	
	Unadjusted PR (95%CI)	Adjusted PR (95%CI)	Unadjusted β (95%CI)	Adjusted β (95%CI)
Breastfeeding (definition A)				
Never breastfed	Ref	Ref	Ref	Ref
Breastfed for <12 months	0.77 (0.52; 1.17)	0.94 (0.63; 1.41)	0.72 (−1.02; 2.65)	1.31 (−0.66; 3.30)
Breastfed for 12 to 23 months	1.11 (0.66; 1.89)	1.47 (0.85; 2.54)	0.88 (−2.00; 3.76)	2.24 (−0.74; 5.24)
Breastfed for 24 months or longer	1.70 (1.09; 2.64)	2.06 (1.35; 3.13)	4.31 (1.19; 7.43)	5.22 (2.06; 8.39)
Breastfeeding (definition B)				
Never breastfed	Ref	Ref	Ref	Ref
Breastfed for <24 months, exclusively until 6	1.00 (0.52; 1.17)	1.45 (0.92; 2.30)	0.70 (−1.85; 3.24)	2.04 (−0.62; 4.71)
Breastfed for <24 months, not exclusively until 6	0.77 (0.66; 1.89)	0.90 (0.59; 1.36)	0.77 (−1.23; 2.77)	1.26 (−0.79; 3.31)
Breastfed for 24 months or longer	1.70 (1.09; 2.64)	2.06 (1.35; 3.13)	4.31 (1.19; 7.43)	5.22 (2.05; 8.39)

Prevalence ratios obtained from Poisson regressions. Beta coefficients obtained from linear regressions. Models adjusted for child: Child sex, household main income source, maternal education at childbirth, index of relative socioeconomic advantage and disadvantage, alcohol consumption in pregnancy, smoking in pregnancy, parity, maternal ethnicity, and whether the child received the intervention (early, delayed).
